# Peptide-Based Regulation of TNF-α-Mediated Cytotoxicity

**DOI:** 10.3390/biom15040559

**Published:** 2025-04-10

**Authors:** Betul Zehra Temur, Ahmet Can Timucin, Ahmet Emin Atik, Tanil Kocagoz, Ozge Can

**Affiliations:** 1 Department of Medical Biotechnology, Institute of Health Sciences, Acibadem Mehmet Ali Aydinlar University, Atasehir, 34752 Istanbul, Turkey; betulzkarakus@gmail.com (B.Z.T.); tanilkocagoz@gmail.com (T.K.); 2 Department of Molecular Biology and Genetics, Faculty of Engineering and Natural Sciences, Acibadem Mehmet Ali Aydinlar University, Atasehir, 34752 İstanbul, Turkey; ahmet.timucin@acibadem.edu.tr; 3 Graduate School of Natural and Applied Sciences, Acibadem Mehmet Ali Aydinlar University, Atasehir, 34752 İstanbul, Turkey; 4 Turgut Ilaclari A.S., 41400 Kocaeli, Turkey; aatik@turgutilac.com.tr; 5 Department of Natural Sciences, Faculty of Engineering and Natural Sciences, Acibadem Mehmet Ali Aydinlar University, Atasehir, 34752 Istanbul, Turkey; 6 Departmen of Medical Microbiology, School of Medicine, Acibadem Mehmet Ali Aydinlar University, Atasehir, 34752 Istanbul, Turkey; 7 Department of Biomedical Engineering, Faculty of Engineering and Natural Sciences, Acibadem Mehmet Ali Aydinlar University, Atasehir, 34752 Istanbul, Turkey

**Keywords:** TNF-α, TNF-α receptors, TNF-α-binding peptide, TNFR1-binding peptide, TNFR2-binding peptide, TNF-α inhibition, TNF-α blocker

## Abstract

Tumor necrosis factor alpha (TNF-α) is a pro-inflammatory cytokine associated with TNF receptor 1 (TNFR1) and TNF receptor 2 (TNFR2), which play important roles in several inflammatory diseases. There is a growing interest in developing alternative molecules that can be used as TNF blockers. In this study, we focused on TNF-α-, TNFR1-, and TNFR2-mimicking peptides to inhibit TNF-α receptor binding in various ways. Six peptides (OB1, OB2, OB5, OB6, OB7, and OB8) were developed to bind TNFR1, TNFR2, and TNF-α. OB1 and OB2 bound to TNF-α with lower *K*_d_ values of 300 and 46.7 nM, respectively, compared to previously published sequences. These synthetic peptides directly and indirectly inhibited TNF-α in vitro without cytotoxicity to L929 cells, and OB1 significantly inhibited apoptosis in the presence of hTNF-α. Peptides developed in this study may prove to be useful for therapeutic inhibition of TNF-α.

## 1. Introduction

TNF-α is a pro-inflammatory cytokine involved in several inflammatory diseases, notably rheumatoid arthritis, asthma, cancer, and neurodegenerative diseases such as Alzheimer’s disease [1]. TNF-α is produced by several cell types, including macrophages, monocytes, T-cells, smooth muscle cells, adipocytes, and fibroblasts, in response to endotoxins or other stimuli [2,3]. TNF-α is a type II transmembrane protein and is converted to soluble TNF-α by TNF-α-converting enzyme (TACE) [4,5]; its molecular weight is 17 kDa, and it is biologically active in vivo as a homotrimer [2].

TNF-α binds with high affinity to two specific receptors on the cell membrane, TNFR1 (p60) and TNFR2 (p80), to exert most of its activity [1]. TNFR1 is more abundant and expressed by all cell types, whereas TNFR2 is present on mainly immune cells [6,7,8]. TNF-α has primarily been associated with the induction of apoptosis, but it can also have the opposite effect by activating inflammation, cell proliferation, and the immune response [6,9].

As a central biological regulator in critical immune functions, apoptosis, and cell survival, TNF-α and its dysregulation have been linked to several diseases. It is one of the most potent anti-tumor cytokines and has become a promising therapeutic in the treatment of cancer [8,10]. Furthermore, inhibition of TNF-α has considerable therapeutic potential for diseases such as diabetes and autoimmune conditions [11]. Monoclonal antibodies (mAbs) and fusion proteins have been used therapeutically to inhibit TNF-α-induced inflammatory responses. mAbs such as infliximab, etanercept, adalimumab, and certolizumab are anti-TNF-α agents used to treat Crohn’s disease, rheumatoid arthritis, and plaque psoriasis [12,13]. Notably, mAbs can also elicit immune-mediated side effects despite their use in the treatment of many diseases [14,15]. Some of these side effects can be predicted by the target molecule, but unpredictable ones can also occur. In addition to immune responses, the development of secondary autoimmunity leads to an increase in malignancies and infections. Immune-related symptoms of mAb therapy include rash, generalized edema, and cardiac arrest [16,17].

mAbs are widely used in high-income countries to treat non-communicable diseases. In contrast, low- and middle-income countries have limited access to existing mAbs, and investment in the development of mAbs is limited due to high costs and the need for advanced laboratory equipment [18]. Long and complex production processes for mAbs increase their costs [13,19]. Alternative therapeutic approaches, particularly peptide-based therapies, may be able to overcome these barriers.

Peptide-based TNF-α inhibition has garnered attention in recent years, as studies have shown that peptides may help treat inflammatory diseases. In a study conducted in 2023, a nine amino acid-long peptide targeting TNF-α was discovered using a T7 phage display library. The pep2 (ACHAWAPTR, K_d_ = 5.14 μM) peptide was shown to bind directly to TNF-α, inhibit TNF-α-induced signaling activation, and reduce inflammation by attenuating NF-κB and MAPK signaling in various cells [20].

Rinvoq (upadacitinib), a small molecule drug used to treat moderate to severe rheumatoid arthritis, was approved by the U.S. Food and Drug Administration in 2019. Later approvals followed for treatment of several inflammatory and autoimmune diseases. Notably, this drug is a Janus kinase (JAK) inhibitor that inhibits TNF-α indirectly as an alternative to mAbs [21]. There are other peptide-based TNF-α inhibitors that have been tested in clinical trials. However, due to the pharmacokinetic limitations of these peptides, the risk of immunogenicity, and poor target specificity, there is no peptide-based TNF-α inhibitor in clinical use.

This study focuses on blocking TNF-α activation through the following two different approaches: inhibition of TNF-α and inhibition of TNF-α receptors. To design peptides from a computational perspective, complex structures containing TNFR1, TNFR2, and TNF-α were retrieved from the Protein Data Bank (PDB). After predicting their peptides and structures with their probable targets using AlphaFold (AlphaFold 3 (default values were used and AlphaFold 2 (alphafold2_multimer_v3 with default values were used)), their protein–peptide heterodimers were then simulated for 100 ns using molecular dynamics (MD), and trajectories were evaluated by estimating their relative binding free energy using the molecular mechanics Poisson-Boltzmann surface area (MM-PBSA) method. Based on computational affinities, three 15-mer peptides, OB5, OB6, and OB8, were suggested to modulate TNF signaling based on their binding affinity for TNFR1, TNFR2, and TNF-α. Three other 20/21-mer peptides, OB1, OB2, and OB7, were designed and synthesized based on reported recognition sequences. After performing solid-phase peptide synthesis (SPPS) of these peptides, the binding of TNF-α-binding peptides and TNFR-binding peptides to their targets was assessed via microscale thermophoresis (MST). We found that these peptides bind to their targets and effectively inhibit TNF-α-mediated cytotoxicity.

## 2. Materials and Methods

### 2.1. Materials

#### 2.1.1. Peptide Synthesis and Analysis

All chemicals and reagents were of analytical grade unless otherwise stated. HPLC-grade acetonitrile was purchased from Merck (Darmstadt, Germany). Anhydrous dimethylformamide (DMF), diisopropylcarbodiimide (DIC), Oxyma^®^, piperidine, dichloromethane (DCM), trifluoroacetic acid (TFA), and sodium iodide (NaI, ≥99.5%) were obtained from Sigma-Aldrich (St. Louis, MO, USA). Leucine enkephaline (YGGFL-OH) was purchased from Waters Corporation (Milford, MA, USA). Ultrapure water (18.2 MΩ·cm) was prepared in-house using a Milli-Q water purification system (Merck-Millipore, Darmstadt, Germany).

#### 2.1.2. Binding Affinity Experiments

An RED-NHS 2nd Generation Protein Labeling Kit and Monolith NT.115 premium capillaries were obtained from NanoTemper Technologies (Munich, Germany).

#### 2.1.3. In Vitro Experiments

Mouse fibroblast (L929) cells (cat# CCL-1) and fetal bovine serum (FBS, 30-2020™) were purchased from ATCC (Manassas, VT, USA). Dulbecco’s modified Eagle’s medium (DMEM) and penicillin–streptomycin (P/S) were purchased from Gibco™ (Grand Island, NY, USA). Human TNFR1 (soluble) recombinant protein and human TNFR2 (soluble) recombinant protein were purchased from PeproTech (Cranbury, NJ, USA).

Pierce™ Quantitative Fluorometric Peptide Assay (Thermo Scientific™, Waltham, MA, USA), MTT cell proliferation kit (Cat. No. 11 465 007 001, Roche, Basel, Switzerland), actinomycin D (Sigma-Aldrich, St. Louis, MO, USA), TNF-α (3rd WHO International Standard, NIBSC, Hertfordshire, UK), human TNF-α antibody (Research Grade Adalimumab Biosimilar) (R&D Systems, Minneapolis, MN, USA), and a Caspase-Glo 3/7 assay system (Promega, Madison, WI, USA) were also used.

### 2.2. Methods

#### 2.2.1. Generation of Target Protein–Peptide Complexes

All necessary dimeric crystal structures were retrieved from PDB, and their sequences that contributed most to binding were predicted using the Rosetta Peptiderive [22] web server. Depending on the target, one interactor was defined as a receptor, and the other partner was used to predict the peptide sequence, whose length was restricted to 15 amino acids. Next, peptides together with their selected targets were fed into AlphaFold 2 (alphafold2_multimer_v3 with default values were used) (Google DeepMind, Mountain View, CA, USA) [23,24,25] or AlphaFold 3 (default values were used) [26] algorithms to retrieve final docked structures. To analyze AlphaFold results, the local confidence in the amino acids in the predicted structure, the quality of the overall prediction, and the accuracy of the peptide positions relative to each other were measured using the predicted local distance different test (pLDDT) score, the predicted template modeling (pTM) score, and the interface pTM (ipTM) score, respectively. For all metrics, structure predictions with the highest values were used and reported.

#### 2.2.2. MD Simulations and Estimation of Relative Binding Free Energies Between Predicted Peptides and Target Proteins

All MD simulations were completed within the TÜBİTAK TRUBA infrastructure. All protein–peptide complexes selected based on AlphaFold predictions were used to prepare MD-ready complexes via CHARMM-GUI [27,28,29] output. In all systems, 150 mM KCl was used for neutralization. Simulations were run with the NAMD 3.0 program with CHARMM36m parameters to execute the simulations [30,31,32,33]; the TIP3P model was used to model water molecules [34,35]. During all production simulations, the NpT ensemble was used with periodic boundary conditions. Columb interactions were computed with the particle mesh Ewald algorithm. Electrostatic and van der Waals interactions were computed within 12 Å. Pressure was kept at 1 atm using the Nosé–Hoover Langevin method, and Langevin dynamics maintained temperature at 310 K [36,37]. Hydrogen bonds were restrained via the SHAKE algorithm [38]. In production runs, coordinates of the system were recorded at every 100 ps.

First, all systems were minimized via the conjugate gradient method for 10,000 steps. Equilibration was completed through the canonical ensemble within 125,000 steps. For each system, production runs were kept at 100 ns. After assessing conformational stability based on root mean square displacement (RMSD) of the backbone heavy atoms (C, O, Cα, and N), the last 10 ns of all simulations that displayed common conformational stability were fed into an MM-PBSA-based estimation of relative binding free energy between peptides and protein targets. For all MM-PBSA calculations, the internal and external dielectric constants were set to 1 and 80, respectively. The entropic term was estimated using quasi-harmonic approximation. A total of 100 snapshots extracted from trajectories were included in calculations. The final relative binding free energies and the enthalpic contributions were estimated using MM-PBSA.py in AmberTools23 [39]. CHARMM parameters were transformed to AMBER via the CHAMBER command available in the topology editor of the ParmEd version of AmberTools23 [40].

#### 2.2.3. Peptide Synthesis

Peptides were synthesized by SPPS, as described by Merrifield, using a CEM Liberty ™ Blue and CEM Discover ™ (Matthews, NC, USA) peptide synthesizer. Rink amide (0.70 mmol/g loading capacity) resin was used in the synthesis. Functional peptides (Table 1) were synthesized as described by Bulut et al. [41].

Three of the peptides used in this study were designed by molecular modeling. These peptides, OB5, OB6, and OB8, have 15 amino acids. The other three were designed by modifying sequences based on the previously published literature. OB1, OB2, and OB7 have 20 amino acids. The ’HIHDDLLRYYGW’ peptide sequence, the foundation for OB1, was originally identified using phage display technology by Brunetti et al. [42]. Although the authors synthesized the sequence in a tetrameric form, we retained the sequence in its monomer form and introduced a lysine residue along with a ‘GGGSGGGS’ linker at the N-terminus of our peptide. This modification was implemented to facilitate binding to an immobilized surface, thereby establishing a foundation for developing novel methods for TNF-α capture. The ‘GGGSGGGS’ linker is a glycine-rich sequence often used to increase peptide solubility. In addition, we standardized the size of these peptides by extending them to a comparable length. Extending the sequence also increased peptide stability. Longer, more flexible linkers also position the peptide more effectively for interaction.

The binding sites of TNFR1 and TNFR2 to TNF-α have been previously studied and characterized. The loop structure of TNFR1 (residues Arg^77^ to Gly^81^) has been reported to interact with TNF-α over a large surface area through van der Waals contacts [43,44,45]. These receptor-ligand binding interactions suggest that the binding region of TNFR1 could be a promising template for developing a potential TNF-α inhibitor. Based on this structural information (RKEMGQV), OB2 (KGGGSGGGSGGGSRKEMGQV) was designed like OB1.

OB7 (VLLTHTISRIAVSYQTKVNLL) is part of the TNF-α sequence that interacts with TNFR1. Key residues in the TNF-α sequence identified for binding to TNFR1 are Ala^84^, Val^85^, Ser^86^, Tyr^87^, Gln^88^**,** and Thr^89^ [46]. Thr^77^, His^78^, Thr^79^, Ser^81^, Pro^90^, Val^91^, Asn^92^, and Leu^93^ are also key to TNF-α-TNFR1 binding [46].

#### 2.2.4. Peptide Purification and Characterization

All synthesized peptides were dissolved in Milli-Q water for further experiments. Synthesized peptides were purified using an AdvanceBio Peptide Plus column (675950-902, Agilent, Santa Clara, CA, USA) with the following two different mobile phases: eluent A (0.05% TFA in H_2_O) and eluent B (0.25% TFA in acetonitrile). Liquid chromatography-tandem mass spectrometry (LC-MS/MS) analysis was performed using an ultraperformance liquid chromatography instrument (Acquity H-Class Bio UPLC) coupled with a Xevo^®^ G2-XS QToF mass spectrometer (Waters, Milford, MA, USA). The instrument was equipped with an electrospray ionization (ESI) source and operated with MSE functionality in positive ion mode. The mass range was set to *m*/*z* 50–2000 for MS and MS/MS analysis. System control, data acquisition, and data processing were carried out using UNIFI^TM^ software (version 1.8.2). Before the analysis, the instrument was calibrated externally using a sodium iodide solution (2 μg/μL). During data collection, reference mass solution (leucine enkephaline, *m*/*z* 556.2766) was continuously infused into the system to correct calibration drift. The following ESI settings were used: capillary voltage 2 kV, cone voltage 50 V, source temperature 100 °C, desolvation temperature 300 °C, sampling cone gas flow 50 L/h, and desolvation gas flow 600 L/h. The scan time for MS/MS was 0.5 s for both precursor and fragment ions [47].

A total of 10 µL of purified peptide sample was injected into the reversed-phase C18 column (Waters Acquity UPLC Peptide CSH C18, 130 Å, 1.7 µm, 2.1 × 100 mm, PN: 186006937). Mobile phases A, B, and C were ultrapure water, acetonitrile, and 1.0% formic acid in ultrapure water, respectively. Separation was performed at a flow rate of 0.2 mL/min in a 25 min linear gradient program, beginning with 5% B for 2 min, followed by 70% B for 14 min, 90% B for 4 min, and column reconditioning for 5 min with 5% B. The column temperature was set to 40 °C, and the eluted peptide was monitored by UV detection at 214 nm.

Peptides were identified by comparing molecular mass and MS/MS fragment ions with the expected amino acid sequence of the peptide. With the help of MS^E^ mode, precursor and fragment ions were collected instantaneously by switching the collision energy between low and high values, respectively, in the collision cell of the mass spectrometer. The mass tolerance for the correct peptide assignment was set to 8 ppm with at least three confirmation fragment ions.

#### 2.2.5. Microscale Thermophoresis

MST was performed using a Monolith NT.115 Pico instrument (NanoTemper Technologies, Munich, Germany). In this technique, thermophoresis applied by irradiation with an infrared (IR) laser diode disrupts a ligand-target binding interaction that has equilibrated. The concentration of fluorescent molecules can be altered by the direct movement of molecules within a temperature gradient. The effect on the fluorescence signal is monitored continuously over time.

Experiments were performed using the Monolith Protein Labeling Kit RED-NHS 2nd Generation and Monolith NT.115 Premium Capillaries at Pico-RED 80% excitation power and medium MST power with thermophoresis. The labeled molecules were used as the target for the binding experiments as listed in Table 1. Mixtures of labeled molecules and functional peptides or TNFR1 were prepared in PBS. Labeled molecules were maintained at 20 nM for the peptide binding experiments, and the ligand concentrations varied according to the molecule with 16 serial dilutions. The peptide-labeled molecule mixture was loaded into the glass capillaries after 20 min incubation in the dark. Results were analyzed using M.O. Affinity Analysis v.2.3 software [48,49].

#### 2.2.6. Cell-Based Assays

The TNF-α neutralizing activity of designed peptides and adalimumab molecules was compared with cell-based bioactivity assays. L929 cells were cultured in DMEM supplemented with 10% FBS and 1% P/S in a humidified 5% CO_2_ incubator at 37 °C [43].

Cytotoxicity of peptides was determined using the 3-(4,5-dimethylthiazol-2-yl)-2,5 diphenyltetrazolium bromide (MTT) cell proliferation assay. Briefly, 3 × 10^4^ cells in 100 μL were seeded into 96-well tissue culture plates and incubated at 37 °C in a 5% CO_2_ incubator for 24 ± 2 h. Peptide concentrations were diluted from 1 pM to 10 μM and measured in triplicate. The following kit protocol and absorbance were measured at 550 nm and 690 nm using a microplate reader (Gen5 Synergy HT BioTek, Winooski, VT, USA) [47].

In the TNF-α neutralization test, 3 × 10^4^ cells in 100 μL were seeded into 96-well tissue culture plates and incubated at 37 °C in a 5% CO_2_ incubator for 24 ± 2 h. After incubation, cells were stimulated with 2 μg/mL actinomycin D and 1 ng/mL TNF-α in the presence of diluted concentrations of adalimumab and functional peptides ranging from 1 pM to 10 μM for 24 ± 2 h. Cells that were not exposed to TNF-α and peptides were considered 100% viable [50]. TNF-α neutralizing activity was also examined by microscopy in a 24-well plate. Both the cytotoxicity effect and neutralizing activity of the OB1 peptide were visually examined by fluorescence microscopy (Zeiss AxioVert A, Oberkochen, Germany). OB1 and adalimumab were administered at a concentration of 1 μM with 1 ng/mL TNF-α.

Apoptosis was detected by measuring caspase-3 and caspase-7 activities with the Promega Caspase-Glo 3/7 Assay Kit. A total of 1.5 × 10^4^ cells were seeded onto opaque 96-well tissue culture plates. After 24 ± 2 h of incubation, cells were stimulated with the same concentration of actinomycin D and TNF-α with different peptide concentrations. After 4 h of incubation in 5% CO_2_ at 37 °C, freshly prepared caspase 3/7 reagent was added to each well, followed by incubation for 1 h at room temperature. Luminescence was measured using a plate reader (CLARIOstar Plus, BMG Labtech, Ortenberg, Germany) [51,52]. Fold change was calculated from the ratio of luminescence of cell control to that of TNF-α-treated cells.

## 3. Results

### 3.1. Computational Prediction of Peptides and Dimeric Protein–Peptide Structures

The Rosetta Peptiderive [22] server was used to predict linear continuous 15-amino acid-long sequences from protein–protein interaction complexes. To predict TNFR1-binding peptide (OB5), asymmetric mouse TNF-α-human TNFR1 complex (PDB ID: 7KP7 chains A and E) was used, and human TNFR1-binding peptide was derived from mouse TNF-α (NHQVEEQLEWLSQRA–TNFR1-binding peptide (OB5)). To predict a peptide that can bind to TNFR2, the crystalline structure of the TNF–TNFR2 complex (PDB ID: 3ALQ chains A and R) was used, and a peptide capable of binding TNFR2 was derived from human TNF (NPQAEGQLQWLNRRA–TNFR2 binding peptide (OB6)). Finally, the tetranectin-derived antagonist–TNF-α complex (PDB ID: 3L9J chains C and T) was utilized to predict a peptide sequence capable of binding TNF-α (KRWSRYFWVDMTGTR–TNF-α-binding peptide (OB8)).

**Figure 1:**
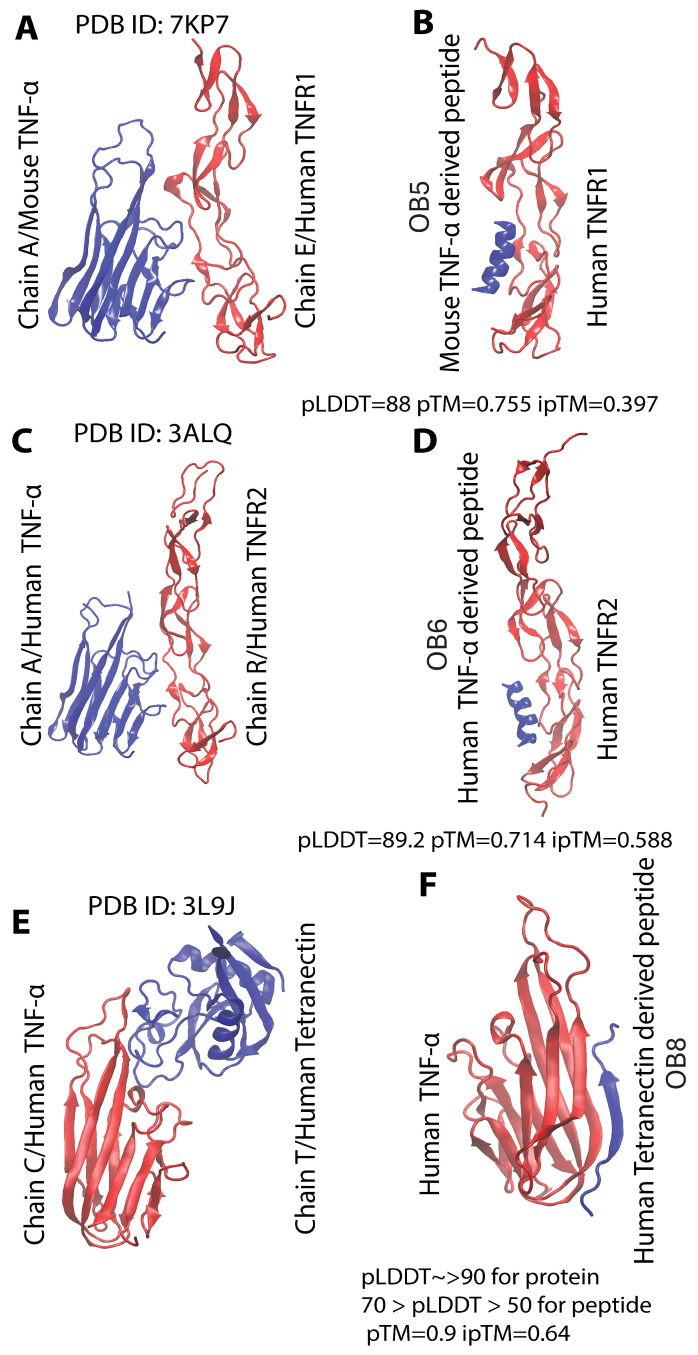
Structures of protein–protein interactions that gave rise to OB5, OB6, and OB8 peptides and target-protein structures retrieved through AlphaFold. (**A**,**C**,**E**) Structures from which the peptides were predicted. (**B**,**D**,**F**) Structure of the AlphaFold 2 (**B**,**D**)- and AlphaFold 3 (**F**)-derived complexes with cartoon representation of the backbone. For all AlphaFold models, the model with the highest pLDDT, pTM, and iPTM scores was selected. As ipTM values were relatively low for the model, MD simulations were then conducted, followed by comparing MM-PBSA-derived relative binding free energy values between peptide and target with MM-PBSA-derived relative binding energy values of the positive control.

Following identification of possible peptide sequences, AlphaFold2 [23,24,25] and AlphaFold3 [26] were used to assemble protein–peptide complexes to be investigated through MD simulations (Figure 1B,D,F). Among AlphaFold-predicted structures, those with higher pLDDT, pTM, and iPTM scores were selected as the most plausible. However, upon further investigation, confidence levels in the estimation of all peptide structures were low, even when the total scores reflect higher confidence levels because the target protein was larger than the peptide. Lower levels of iPTM scores in all AlphaFold-predicted structures prompted us to characterize the stability of these complexes in solution. Thus, 100 ns MD simulations were conducted for all complexes shown in Figure 1; the affinities of peptide-containing complexes were then compared with the original complexes from which the peptides were derived.

### 3.2. MD Simulations and Estimation of Relative Binding Free Energies Between Predicted Peptides and Target Proteins

**Figure 2:**
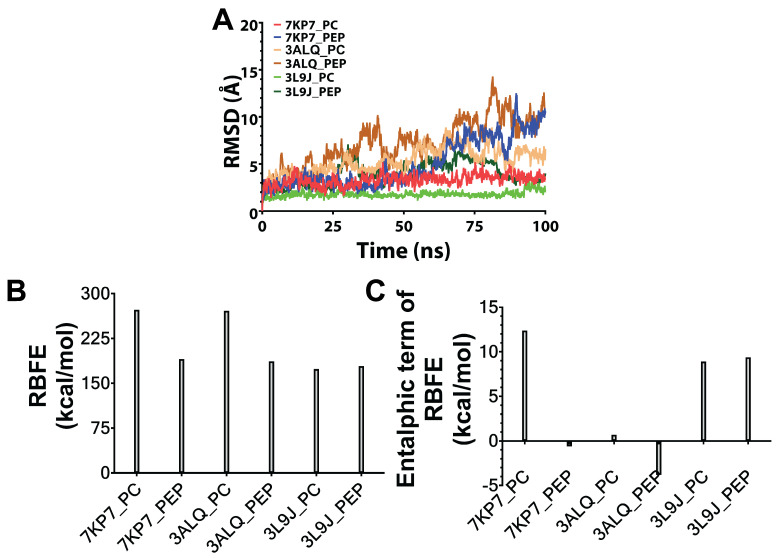
Conformational stability, total relative binding free energy (RBFE), and enthalpic contribution to RBFE retrieved from MD simulations of the structures. (**A**) RMSD-based conformational stability analyses of 100 ns MD simulations indicated that the last 10 ns of each simulation could be considered stable. Thus, a trajectory encompassing the last 10 ns was used for estimating RBFE for each simulation using the MM-PBSA method. (**B**) Based on RBFE analyses, the affinities of peptides capable of binding TNFR1 (peptide in the 7KP7_PEP complex) and TNFR2 (peptide in the 3ALQ_PEP complex) were higher than those of their corresponding positive control complexes (7KP7_PC and 3ALQ_PC). TNF-α-binding peptide (peptide in the 3L9J_PEP complex) also returned comparable affinity with its positive control complex (3L9J_PC). (**C**) Given that the entropic term was estimated via error-prone quasi-harmonic approximation, the enthalpic contribution to RBFE was also computed and shown to have a similar pattern.

After each complex in Figure 1 underwent a 100 ns MD simulation, conformational stability was assessed based on RMSD measurements of the backbone atoms. These analyses revealed that at least the last 10 ns of all simulations presented conformational stability (Figure 2A); thus, this time period was used to estimate the relative binding free energy between peptides and their targets. According to MM-PBSA-based estimation of relative free energy binding levels, TNFR1- and TNFR2-binding peptide-containing complexes had higher affinity than their native positive control complexes, whereas the TNF-binding peptide-containing complex had only comparable affinity with its corresponding positive control (Figure 2B). In addition, the enthalpic contribution to the total energy level was estimated given that the quasi-harmonic approximation of the entropic term was prone to high error rates (Figure 2C). Complementing the estimations of relative binding free energy, enthalpic contribution showed the same trend. Based on these results, these computationally identified peptides were used as potential modulators of TNF signaling.

### 3.3. Synthesis and Characterization of Designed Peptides

**Figure 3:**
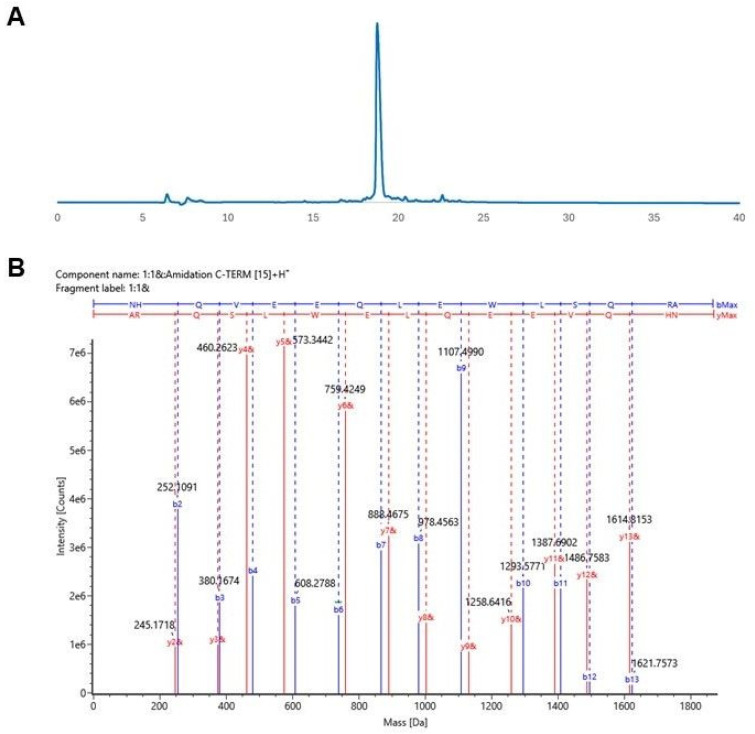
Characterization of OB5 peptide: (**A**) HPLC chromatogram and (**B**) MS/MS spectrum.

The peptides listed in Table 1 were synthesized and characterized by liquid chromatography and LC-MS/MS. HPLC chromatograms and MS/MS spectra of peptides are provided in Supplementary Information. The UV chromatogram and mass spectra of the OB5 peptide are shown as an example in Figure 3.

The OB5 peptide has the sequence ‘NHQVEEQLEWLSQRA’, and its HPLC chromatogram is shown in Figure 3A. OB5 was eluted at 18.75 min with a purity of >99%. The molecular ion of the C-terminal-amidated OB5 peptide was detected at *m*/*z* 1866.9030 (expected mass: *m*/*z* 1866.9008) with a mass error of −4.6 ppm. The acquired MS/MS spectrum for this peak is presented in Figure 3B.

### 3.4. Binding Affinities of Designed Peptides for Their Target Molecules

**Figure 4:**
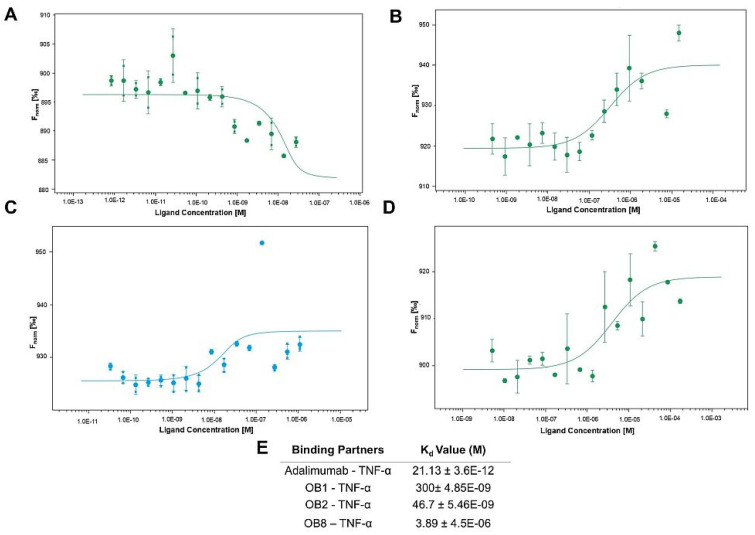
Binding affinities of the peptides for rhTNF-α via microscale thermophoresis. Binding curves of (**A**) adalimumab, (**B**) OB1, (**C**) OB2, and (**D**) OB8. MST experiments were performed using labeled rhTNF-α at 20 nM and variable concentrations of adalimumab and synthesized peptides. TNFR1 and rhTNF-α MST experiments were performed using 1 nM of labeled TNFR1. Binding data are plotted as the mean ± SD of three replicates. (**E**) Dissociation constant (*K*_d_) of indicated interactions given as mean ± SD of three replicates.

**Figure 5:**
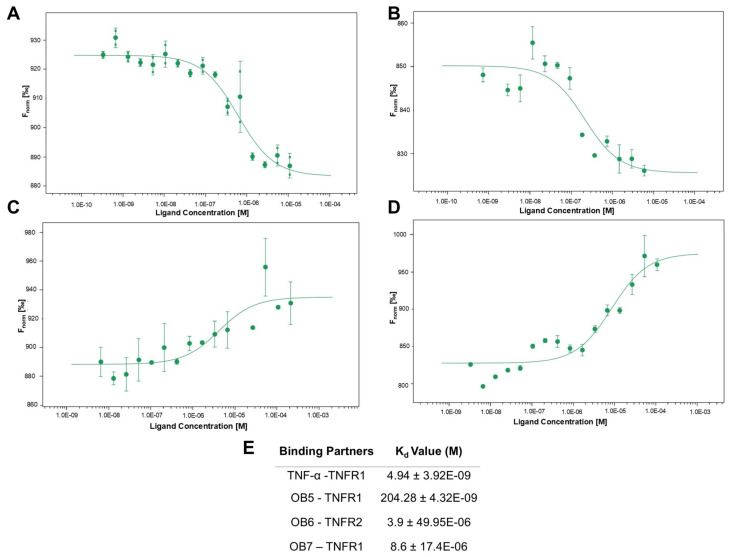
Binding affinities of the peptides for TNFR1 and TNFR2 measured by MST. Binding curves of (**A**) rhTNF-α, (**B**) OB5, (**C**) OB6, and (**D**) OB7. MST experiments were performed using labeled TNFR1 and TNFR2 at 20 nM and variable concentrations of synthesized peptides. TNFR1 and rhTNF-α MST experiments were performed using 1 nM of labeled TNFR1. Binding data are plotted as the mean ± SD of three replicates. (**E**) *K*_d_ values of indicated interactions are presented as mean ± SD of three replicates.

The affinity of the synthesized peptides for their target molecules was evaluated using MST; binding curves are presented in Figure 4 and Figure 5.

Figure 4A shows the binding of adalimumab to rhTNF-α as a standard. Figure 4B–D show the binding of rhTNF-α molecules to OB1, OB2, and OB8, respectively. The experimental *K*_d_ values of these peptides were 300 ± 4.85 nM, 46.7 ± 5.46 nM, and 3.89 ± 4.54 µM, respectively, and adalimumab-rhTNF-α has a *K*_d_ of 21.13 ± 3.6 pM (Figure 4E).

OB5 and OB7 are designed to bind TNFR1, whereas OB6 is designed to bind TNFR2. The interaction between fluorescently labeled TNFR1 and rhTNF-α served as a control (Figure 5A). Figure 5B–D show the binding of fluorescently labeled TNFR1 with OB5, OB6, and OB7, respectively. The calculated *K*_d_ for TNFR1 and OB5 (204.28 nM) indicates a high affinity like that observed for TNFR1-rhTNF-α (Figure 5E).

### 3.5. Inhibition of TNF-α-Mediated Cellular Cytotoxicity and TNFR Binding Using Synthesized Peptides

The L929 cell line has TNF receptors (TNFR1 and TNFR2) located on the cell surface. L929 is a fibroblast mouse cell line that is sensitive to apoptosis caused by stimulation with TNF-α, which initiates caspase-3/7-mediated apoptosis by triggering caspase-8 activation. To inhibit this interaction, we synthesized the following two different peptides: peptides that bind to TNF-α and peptides that bind to TNF receptors.

**Figure 6:**
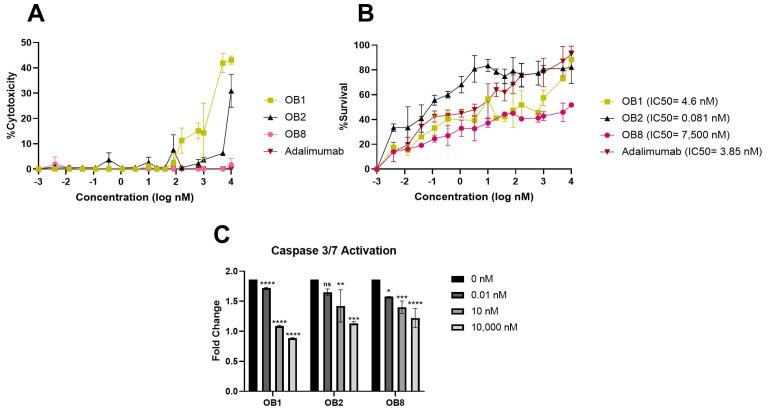
Inhibition of TNF-α activity on L929 cells by TNF-α-binding peptides compared to adalimumab. (**A**) Cytotoxicity of synthesized peptides and adalimumab and (**B**) neutralization of TNF-α-induced cytotoxicity. All data are presented as means ± SEM of triplicate experiments. (**C**) The effect of TNF-α-binding peptides on caspase 3/7 activation. Results are presented as means ± SEM. ns: not significant; * *p* < 0.05; ** *p* < 0.01; *** *p* < 0.001; **** *p* ≤ 0.0001 vs. the control group.

First, we evaluated the cytotoxicity of synthesized peptides at different concentrations on the L929 cell line using the MTT assay. Figure 6A shows the cytotoxicity of the TNF-α-binding peptides (OB1, OB2, and OB8) and adalimumab. OB8 and adalimumab are non-toxic even at a concentration of 10,000 nM. The toxicity of OB1 and OB2 increased over a range of 100 to 10,000 nM, though it did not exceed 50% cytotoxicity even at the highest concentration that corresponded to the maximum level of TNF-α neutralization (Figure 6A,B). Adalimumab reduced TNF-α-induced L929 cell death (IC50 = 3.85 nM measured in our experiments). OB1, OB2, and adalimumab had similar neutralizing activity that corresponded to approximately an 80% survival rate at a concentration of 10,000 nM (IC50 = 4.6 nM for OB1 and IC50 = 0.081 nM for OB2). OB2 showed higher neutralizing activity at lower concentrations compared to other molecules (Figure 6B).

**Figure 7:**
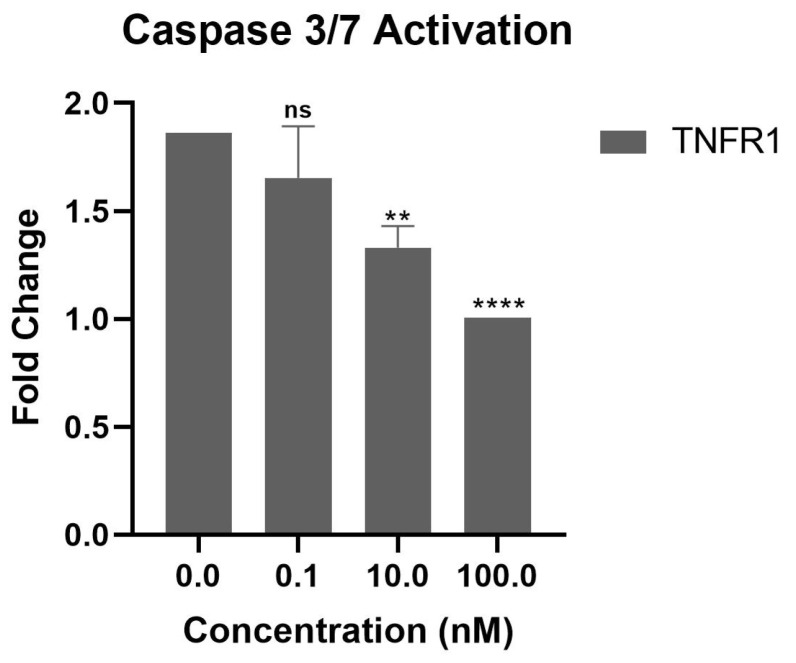
The effect of TNFR1 applied with TNF-α on caspase 3/7 activity. Results are presented as means ± SEM. ns, not significant; ** *p* < 0.01; **** *p* ≤ 0.0001 vs. the control group.

Figure 6C shows that OB1, OB2, and OB8 inhibited TNF-α-induced apoptosis in a concentration-dependent manner compared to TNF-α-applied controls. OB1 was significantly more effective in inhibiting apoptosis. In Figure 7, caspase 3/7 activity exerted on the L929 cell line was evaluated when 100 nM, 10 nM, and 0.1 nM of TNFR1 were applied with the same concentration of TNF-α (1 ng/mL). TNFR1 significantly reduced caspase 3/7 activity at 10 nM and 100 nM. In comparison with TNFR1, OB1 had a similar effect on apoptosis at a 10 nM concentration.

**Figure 8:**
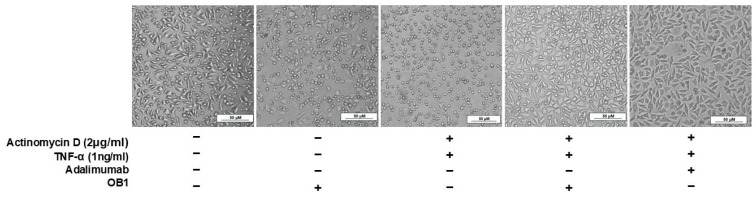
Morphological analysis of L929 cells after treatment with different molecules.

Morphological studies were also performed to explore how OB1 and adalimumab used in the neutralization assays affect L929 cells (Figure 8). It has been shown that the use of TNF-α (1 ng/mL) and actinomycin D together causes almost 100% cell death, whereas no cell death was observed in the presence of the OB1 peptide. The presence of OB1, or adalimumab, with TNF-α (1 ng/mL) and actinomycin D enhanced cell survival.

**Figure 9:**
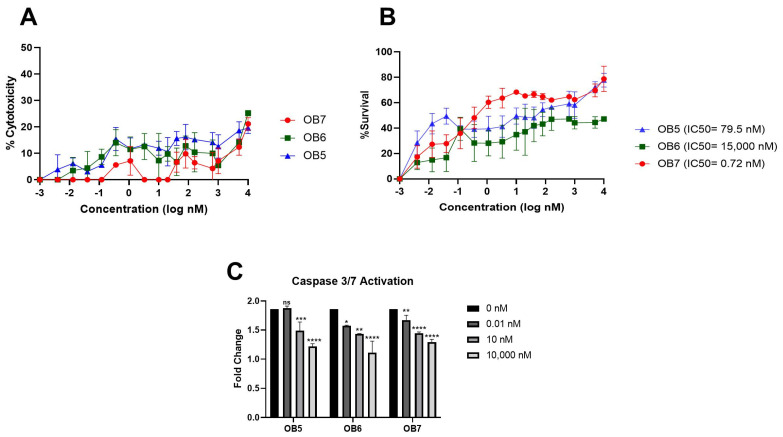
Inhibition of TNF-α activity on L929 cells by receptor-binding peptides. (**A**) Cytotoxicity of synthesized peptides and (**B**) neutralization of TNF-α-induced cytotoxicity measured by MTT assay. All data are presented as means ± SEM of triplicate experiments. (**C**) The effect of receptor-binding peptides on caspase 3/7 activation. Results are presented as means ± SEM. ns: not significant; * *p* < 0.05; ** *p* < 0.01; *** *p* < 0.001; **** *p* ≤ 0.0001 vs. the control group.

Cytotoxicity of the receptor-binding peptides increased in a dose-dependent manner, yet their toxicity remained below 50% even at a concentration of 10,000 nM (Figure 9A). OB5 and OB7 are expected to bind to TNFR1, and OB6 binds to TNFR2 to block the binding of TNF-α and enhance cell survival. OB7, which is designed to bind TNFR1, showed better neutralizing activity (IC50 = 0.72 nM) than OB5 and OB6 (Figure 9B). OB6 and OB8, despite having different targets (TNFR2 and TNF-α, respectively), showed similar TNF-α neutralization activity (Figure 6B and Figure 9B). Based on the caspase 3/7 activity assay, OB5, OB6, and OB7 had similar concentration-dependent caspase activities; despite its lower neutralizing activity, OB6 inhibited apoptosis more than OB5 and OB7 (Figure 9C).

## 4. Discussion

TNF-α orchestrates the progression of autoimmune disease by inducing apoptosis or by activating the NF-κB pathway for inflammatory gene activation; it exists in either soluble or membrane-anchored form, both of which can interact with TNFR1 and TNFR2 to induce inflammatory signaling cascades [53]. Given safety and economic concerns associated with mAb-based therapies for treatment of autoimmune conditions, this study investigated the potential of peptide-based TNF-α inhibitors as alternative therapeutic agents. Here, we computationally identified three peptides (OB5, OB6, and OB8) that likely modulate the TNF signaling pathway and could have therapeutic value for tissue regeneration, wound healing, and treatment of autoimmune diseases.

The three TNF-α-binding peptides synthesized in this study, OB1, OB2, and OB8, have different binding affinities and TNF-α neutralization efficiencies. OB1 had a strong affinity for TNF-α (*K*_d_ = 300 nM), consistent with its potent inhibition of TNF-α (IC50 = 4.6 nM) (Figure 6B). OB1, with its high affinity for TNF-α, neutralized TNF-α by preventing its interaction with TNFR1, thereby serving as an antagonist in TNF-α signaling. OB8, a novel peptide designed in this study, had a binding affinity of 3.89 μM (Figure 4E) and an IC50 of 7500 nM, a significantly lower neutralizing capacity compared to OB1 and OB2.

The mechanism of TNF-α binding distinguishes these peptides from direct TNFR antagonists in that they inhibit TNF-α rather than directly blocking TNFR function. Although all three peptides directly bound to TNF-α, they competed with TNFR1, reducing the availability of free TNF-α and thus inhibiting activation of TNFR1. This suggests that they may prevent TNF-α from effectively activating TNFR1. OB1, with its high affinity for TNF-α, effectively prevented TNFR1 activation. OB1’s strong inhibition of TNF-α-induced cell death supports this hypothesis. Moreover, OB2 had a superior neutralization efficiency despite its lower binding affinity, and it effectively reduced the activation of caspase-3/7. However, OB8 had a lower effect on caspase activation, consistent with its weaker neutralization of TNF-α.

Adalimumab is one of the most potent inhibitors of TNF-α in clinical use today. It directly binds to sTNF-α, thereby blocking its binding to cell surface receptors TNFR1 and TNFR2 [53]. The *K*_d_ of adalimumab–TNF-α was found to be 21.13 pM, which is a considerably high affinity (Figure 4A,E). OB1 is a strong candidate for TNF-α neutralization (IC50 = 4.6 nM), performing similarly to adalimumab (3.85 nM) (Figure 6B).

The sequence ‘HIHDDLLLRYYYGW’ was synthesized in tetrameric form by Brunetti et al., who reported that their tetra-branched anti-TNF-α had a *K*_d_ of 6 μM [42]. Using the monomer and extended version of their peptide here, OB1 had a *K*_d_ of 300 ± 4.85 nM (Figure 4B,D) and inhibited TNF-α by more than 80% at a concentration of 10 μM (IC50 = 4.6 nM) without cytotoxicity against L929 cells (Figure 5B). OB1 also attenuated TNF-α-induced cell death based on morphological examination (Figure 8). Brunetti et al. showed that tetra-branched anti-TNF-α inhibited TNF-α–TNFR2 interaction with an IC50 of 4.3 μM [42], whereas OB1 inhibited TNF-α with an IC50 of 4.6 nM. This difference of three orders of magnitude can be explained by differences in methods; here, OB1’s IC50 was measured in a cell-based assay, whereas the tetra-branched peptide’s IC50 was measured using surface plasmon resonance. The tetra-branched peptide structure is widely used due to its stability against proteases and peptidases and long half-life [42]. The peptides presented here should be synthesized in different structural forms to make them protease-resistant and to increase their half-life.

The OB2 peptide, which is designed to mimic TNFR1’s binding region, may function similarly to sTNFR1 and has a lower *K*_d_ of 46.7 nM. However, OB2 shows remarkable inhibition of TNF-α with an IC50 of 0.081 nM. This indicates a highly effective neutralization mechanism despite its low affinity. The peptide that mimicked TNFR1 may act like sTNFR1 and be better at neutralizing TNF-α. OB2 can effectively trap TNF-α and prevent its interaction with TNFR1 in a similar way to sTNFR1.

C87 is a small molecule whose docking template is the loop structure of TNR1 from Arg^77^ to Val^83^ (RKEMGQV) that blocks signal transduction of TNFR1–TNF-α by binding TNF-α [50] as the OB2 peptide. Thus, C87 is used as a positive control in many TNF-α inhibitor studies. The binding affinity of C87 was previously measured using SPR with a *K*_d_ of 110 nM. Our peptide OB2, which includes the same loop sequence, had a *K*_d_ of 46.7 nM. The IC50 of C87 for TNF-α-mediated cytotoxicity was 8.73 μM, whereas that of OB2 was 0.081 nM. Although C87 binds TNF more strongly, OB2 was more effective at inhibiting TNF-α in vivo even at low concentrations (Figure 6B). In contrast, C87 failed to inhibit the interaction of TNF-α with both TNFR1 and TNFR2 in vitro. TNF-α-induced apoptosis is mediated by binding TNF-α to type I receptors and activating the apoptotic cascade. Ma et al. showed that C87 inhibits the TNF-α-induced activation of caspase-3 and -8 [50]. Here, OB2 significantly reduced caspase activation at 10,000 nM.

The sequences for OB5 and OB6 were predicted by molecular docking studies. These peptides target TNFR1 and TNFR2, respectively, which are both sensitive to TNF-α and have a specific binding domain. Based on their computational affinities, we found that OB5 and OB6 tended to bind to their targets. OB5–TNFR1 and OB6–TNFR2 interactions had K_d_ values of 204.2 nM and 3.9 μM, respectively (Figure 5E), though OB5 had a stronger neutralizing effect (IC50 = 79.5 nM) than OB6 (IC50 = 15,000 nM) (Figure 9B). The reduced ability of the OB6 to inhibit TNF-α may be related to the mechanism of TNFR2–TNF-α interaction and signal transduction; TNFR1 efficiently interacts with both soluble and membrane-bound TNF-α (mTNF-α), whereas TNFR2 is mainly activated by mTNF-α [54]. Receptors such as TNFR1 and TNFR2 are normally activated by trimeric TNF-α, but small peptides can bind to specific sites on the receptors and act as agonists or antagonists to activate or block the receptor. Peptides targeting TNFR1 may be more effective in blocking the biological activity of TNF-α by inhibiting interaction with sTNF-α. TNFR2-targeting peptides depend on mTNF-α and therefore may be less effective in neutralizing free TNF-α. OB5 may compete with TNF-α and block TNF-α–TNFR1 binding to reduce apoptotic and inflammatory signaling. However, at high concentrations, OB6 still inhibited 50% of TNF-α activity despite its low affinity for TNFR2. This suggests that OB6 may play an indirect role, possibly by modulating TNFR2 signaling.

The findings of the caspase 3/7 activation assay further support this hypothesis. TNFR1 and TNFR2 are both transmembrane proteins, but only TNFR1 has a death domain and can activate apoptosis. Moreover, TNFR2 lacks this death domain and can activate anti-apoptotic signals to promote cell survival [55]. The fact that OB6 reduced caspase-3 and caspase-7 levels suggests that TNFR2 enhances its anti-apoptotic effects (Figure 9C). The exact mechanism of TNFR2 may indirectly suppress apoptosis through NF-κB or PI3K/Akt signaling, which could explain the reduced caspase 3/7 levels despite low TNF-α neutralization. Further experiments are needed to confirm the exact mechanism by which OB6 suppresses apoptosis.

OB7, a small binding fragment of TNF-α, had a *K*_d_ of 8.6 μM when interacting with TNFR1, whereas the full-length TNF-α–TNFR1 interaction had a *K*_d_ of 4.94 nM (Figure 5D). Accordingly, OB7 had a lower inhibition effect on the apoptosis cascade based on the caspase 3/7 assay results (Figure 9C).

Compared with two TNFR1-binding peptides, OB5 and OB7, OB5 demonstrated stronger affinity for TNFR1 (*K*_d_ = 204.2 nM), whereas OB7 had a weaker affinity (*K*_d_ = 8.6 μM) (Figure 5D). Furthermore, the TNF-α neutralization efficiency of OB7 (IC50 = 0.72 nM) was superior to that of OB5 (IC50 = 79.5 nM). OB7 may effectively compete with TNF-α for TNFR1 binding, thereby blocking apoptotic and inflammatory signaling (Figure 9C). In contrast, OB5 did not fully inhibit TNF-α–TNFR1 interaction, leading to partial rather than full receptor activation. This difference suggests that OB7 may directly interfere with TNFR1 activation. In contrast, OB5 may primarily modulate TNF-α availability rather than completely blocking TNFR1 activation.

Although these peptides are likely functional, there are some limitations to this study. First, it is not clear if the AlphaFold-derived structures comply with other global docking programs. Moreover, the structures of these peptides should be further elucidated to verify the proposed interactions are physically real. Second, the entropic term was estimated using a quasi-harmonic approximation of the MM-PBSA method, and the relative binding free energies were possibly skewed from absolute values. Thus, the binding affinities should be calculated using more sophisticated computational methodologies. Third, it is not clear if these peptides can penetrate the cell solely on their own. The addition of a cell-penetrating peptide to the N- or C-terminus may be necessary.

For treatment of autoimmune diseases, peptide-based inhibitors offer several advantages over mAb-based therapies, including lower production costs and production time. They may penetrate tissues more easily due to their smaller size and reduced immunogenicity. However, significant barriers to clinical development include their limited binding affinity and rapid degradation, leading to short circulation half-life and poor systemic bioavailability [56]. Although this study computationally identified peptides with potential therapeutic benefit, their pharmacokinetics and bioavailability should be characterized in future studies. Overcoming these barriers will be critical for advancing peptide therapeutics into mainstream clinical practice.

## 5. Conclusions

This study highlighted the potential of peptide-based TNF-α inhibitors as viable alternatives to monoclonal antibodies for the modulation of inflammatory and autoimmune responses. The designed peptides showed bound and neutralized TNF-α to varying degrees, with OB1 and OB2 showing the most potent inhibitory effects. OB5 and OB6, targeting TNFR1 and TNFR2, respectively, provided additional insights into receptor-specific regulation, suggesting that peptide-based inhibition of TNF signaling may offer therapeutic benefits. Overall, peptide-based TNF-α inhibitors have significant potential as next-generation therapeutics for inflammatory diseases. Further research focusing on in vivo efficacy, pharmacokinetics, and targeted delivery mechanisms is needed to advance these molecules toward clinical applications.

## Figures and Tables

**Table 1:** List of synthesized peptides.

Peptide	Sequence	Molecular Weight (g/mol)	Binding Partner
OB1	KGGGSGGGSHIHDDLLRYYGW	2231.42	rhTNF-α
OB2	KGGGSGGGSGGGSRKEMGQV	1748.90	rhTNF-α
OB5	NHQVEEQLEWLSQRA	1866.02	TNFR1
OB6	NPQAEGQLQWLNRRA	1779.98	TNFR2
OB7	VLLTHTISRIAVSYQTKVNLL	2368.85	TNFR1
OB8	KRWSRYFWVDMTGTR	1988.31	rhTNF-α

## Data Availability

All the data are included in the manuscript.

## Supplementary Materials

The following supporting information can be downloaded at: https://www.mdpi.com/article/10.3390/biom15040559/s1, Figure S1: (A) HPLC chromatogram and (B) MS/MS spectrum of OB1 peptide; Figure S2: (A) HPLC chromatogram and (B) MS/MS spectrum of OB2 peptide; Figure S3: (A) HPLC chromatogram and (B) MS/MS spectrum of OB6 peptide; Figure S4: (A) HPLC chromatogram and (B) MS/MS spectrum of OB7 peptide; Figure S5: (A) HPLC chromatogram and (B) MS/MS spectrum of OB8 peptide; Figure S6: (A) HPLC chromatogram and (B) MS/MS spectrum of OB6 peptide;Figure S7: MS Spectra of OB6 peptide; Figure S8: (A) HPLC chromatogram and (B) MS/MS spectrum of OB7 peptide; Figure S9: MS Spectra of OB7 peptide; Figure S10: (A) HPLC chromatogram and (B) MS/MS spectrum of OB8 peptide; Figure S11: MS Spectra of OB8 peptide.

## Abbreviations

The following abbreviations are used in this manuscript:

TNF-α	tumor necrosis factor alpha
TNFR1	TNF receptor 1
TNFR2	TNF receptor 2
MST	microscale thermophoresis
TACE	TNF-α-converting enzyme
mAbs	monoclonal antibodies
PDB	Protein Data Bank
pLDDT	predicted local distance difference test
RMSD	root mean square displacement
SPPS	solid-phase peptide synthesis
LC-MS/MS	liquid chromatography-tandem mass spectrometry
K_d_	dissociation equilibrium constant
