# Supplementary material for: Peptide-Based Regulation of TNF-α-Mediated Cytotoxicity
Source: Biomolecules. 2025 Apr 10;15(4):559. doi: 10.3390/biom15040559 (PMC12024540; doi:10.3390/biom15040559)
Supplement: Supplementary file 1 [file biomolecules-15-00559-s001.zip › biomolecules-3538625-supplementary.pdf]

Supplementary Materials

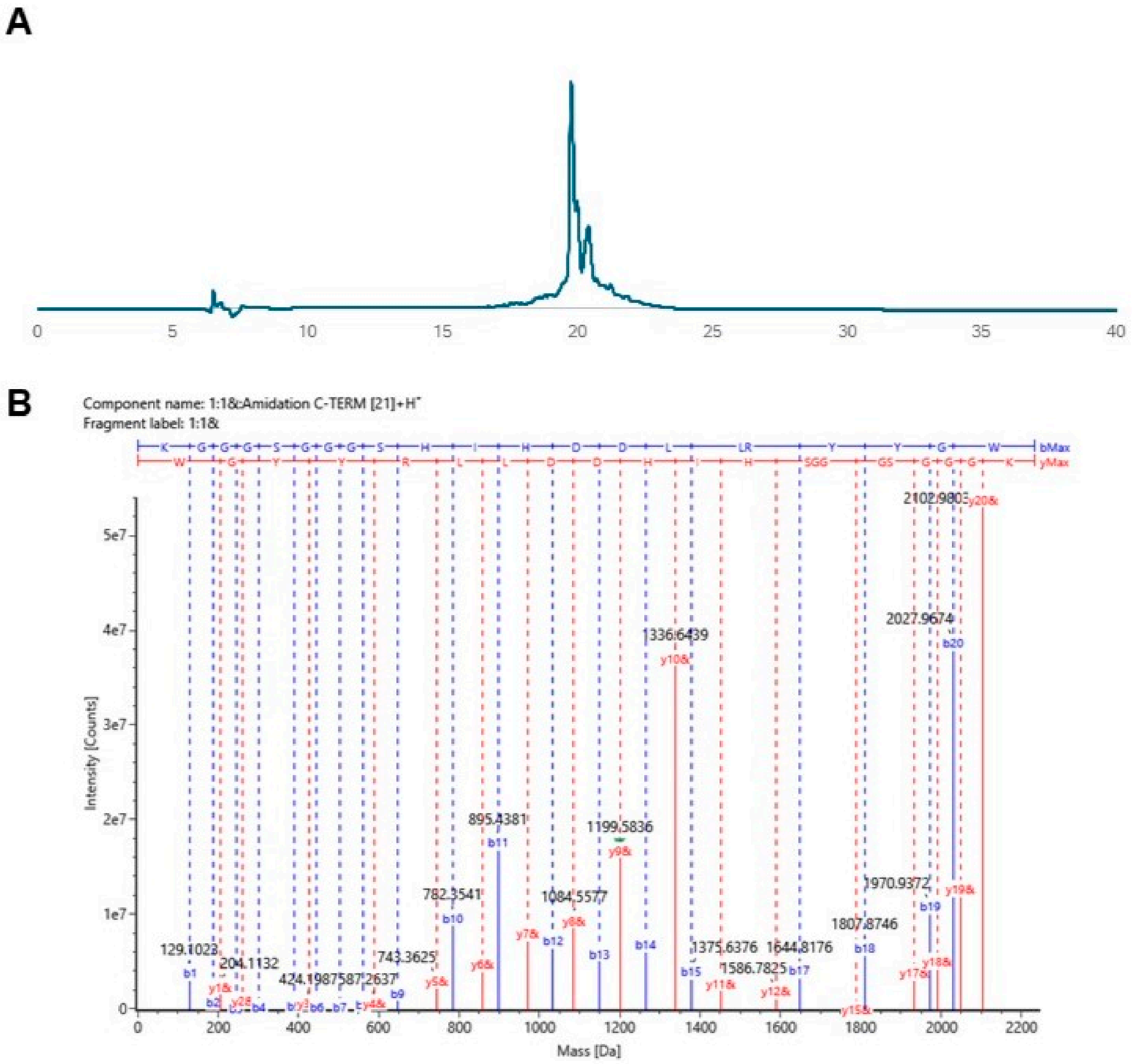

Figure S1. (**A**) HPLC chromatogram and (**B**) MS/MS spectra of OB1 peptide.

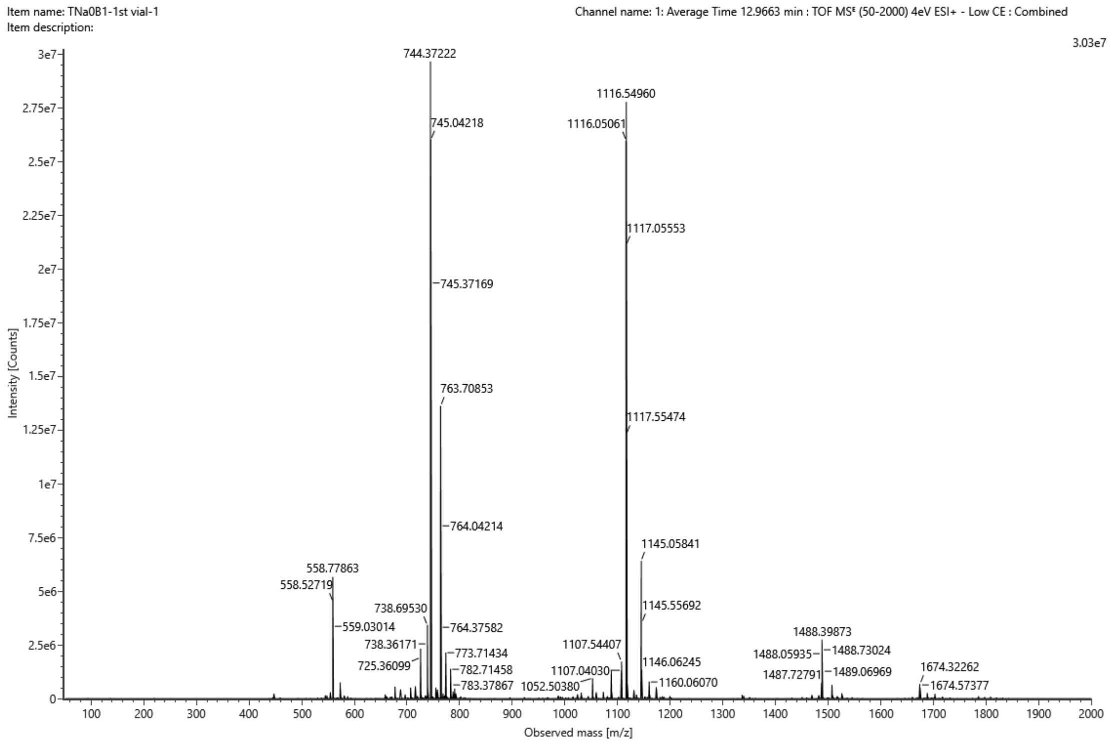

Figure S2. MS Spectra of OB1 peptide.

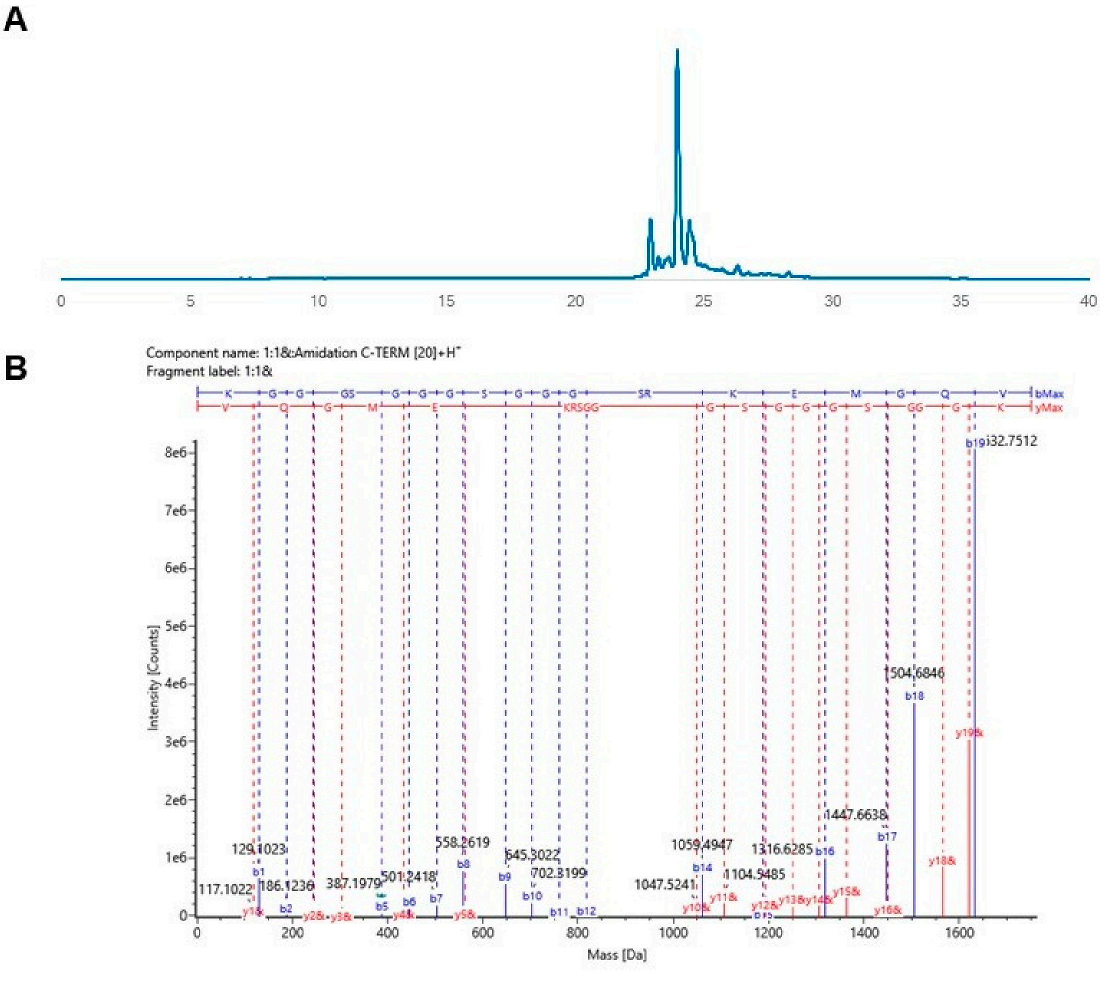

Figure S3. (**A**) HPLC chromatogram and (**B**) MS/MS spectrum of OB2 peptide.

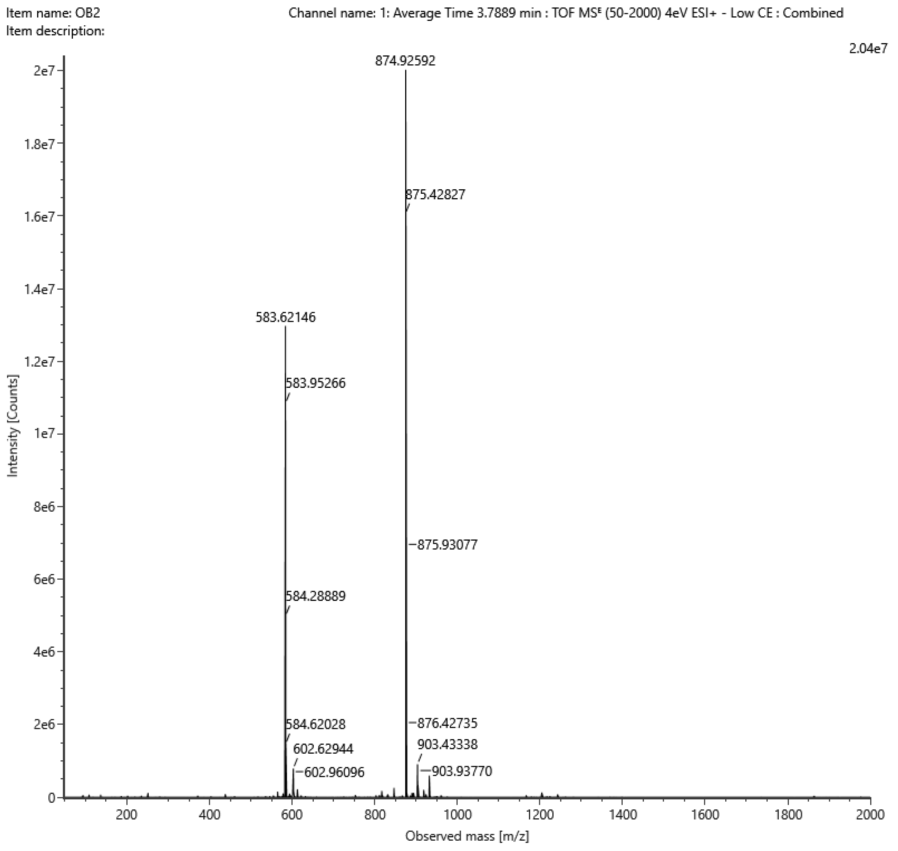

Figure S4. MS Spectra of OB2 peptide.

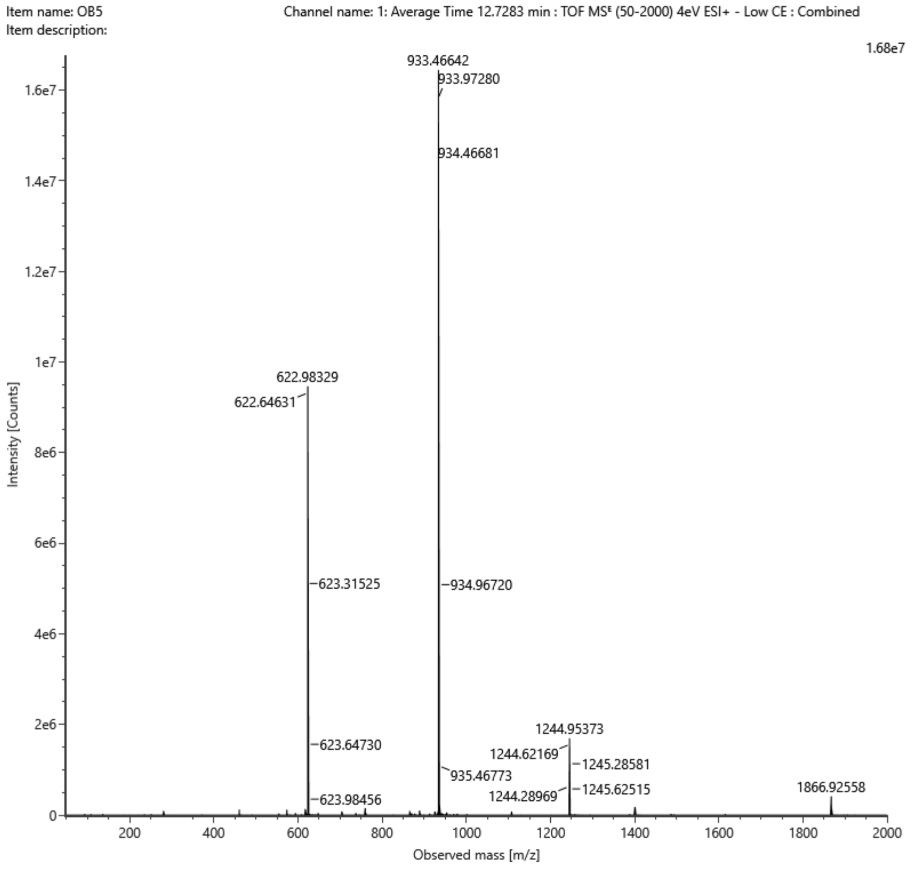

Figure S5. MS Spectra of OB5 peptide.

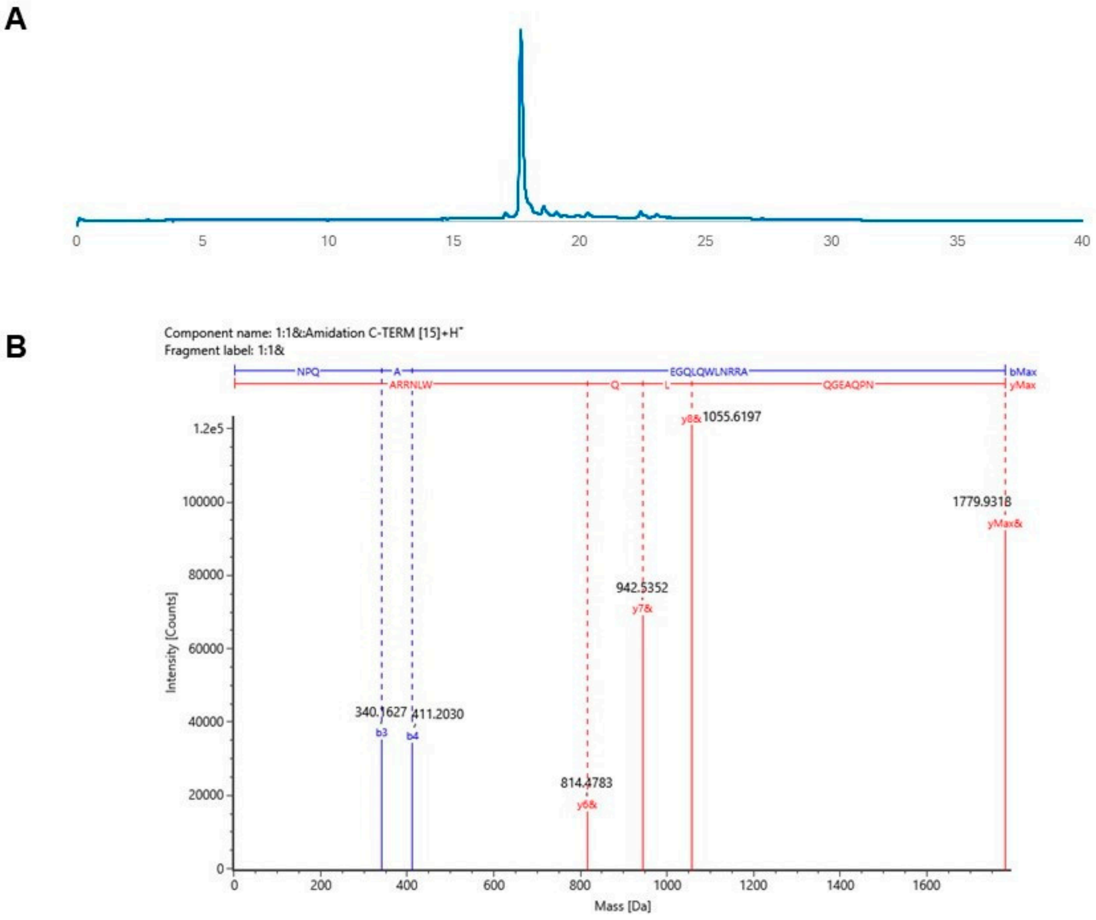

Figure S6. (**A**) HPLC chromatogram and (**B**) MS/MS spectrum of OB6 peptide.

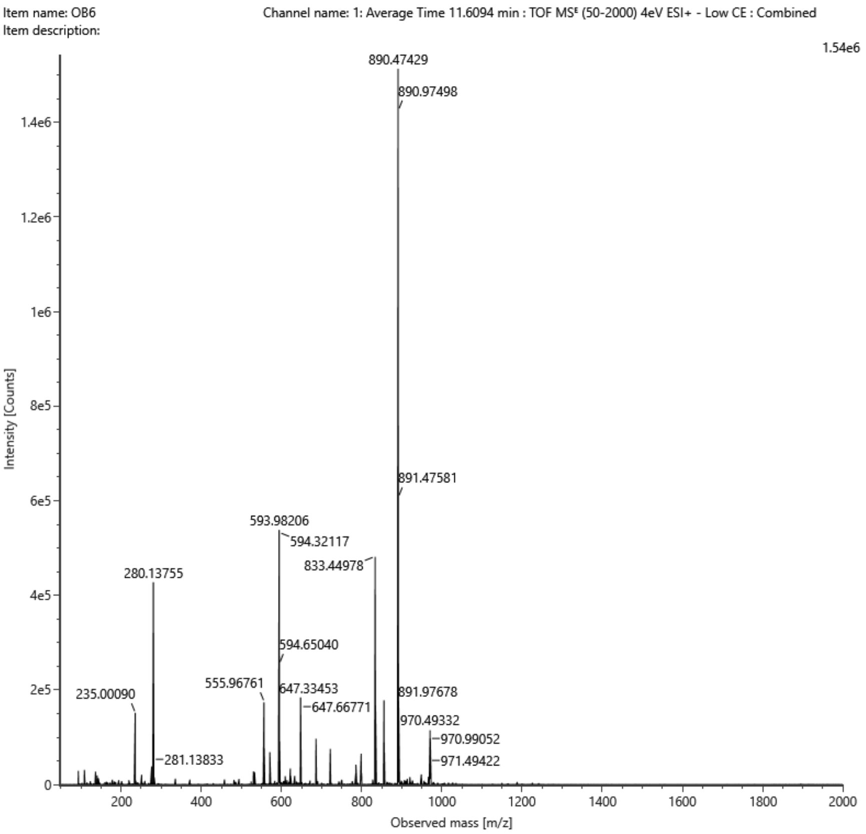

Figure S7. MS Spectra of OB6 peptide.

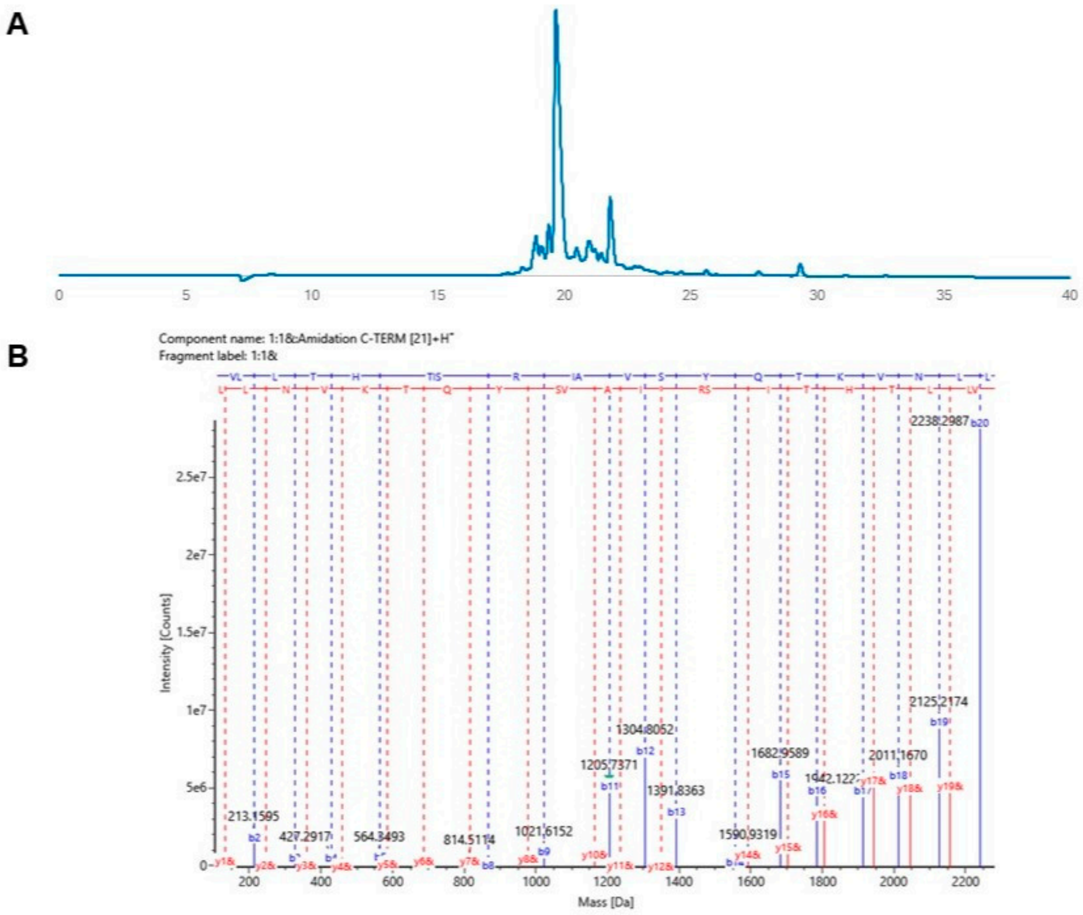

Figure S8. (**A**) HPLC chromatogram and (**B**) MS/MS spectrum of OB7 peptide.

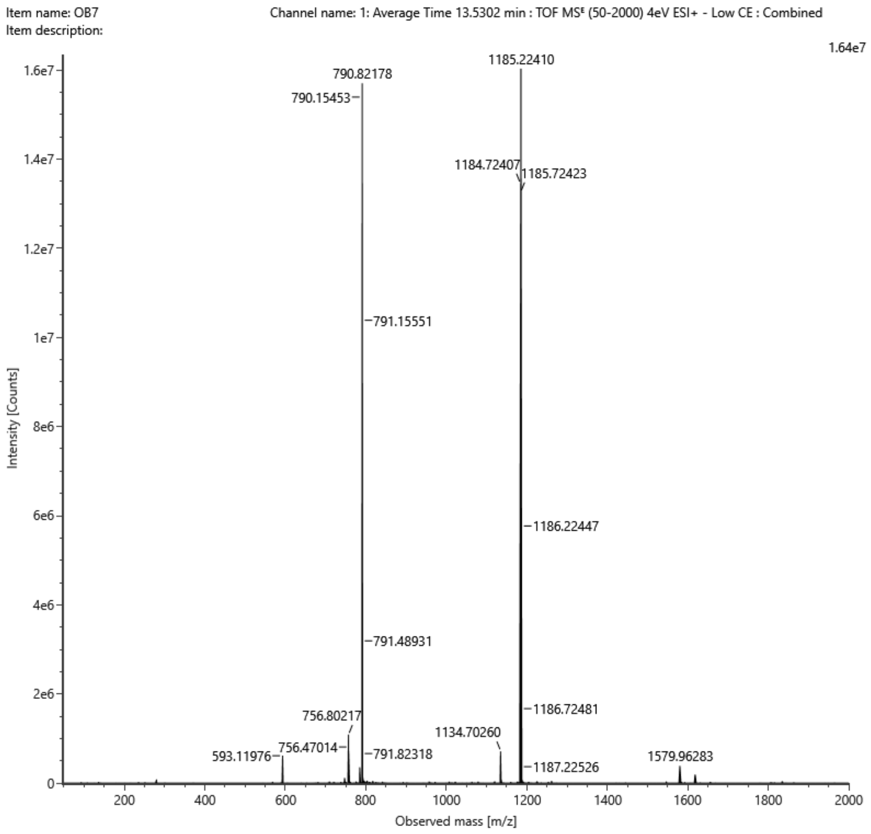

Figure S9. MS Spectra of OB7 peptide.

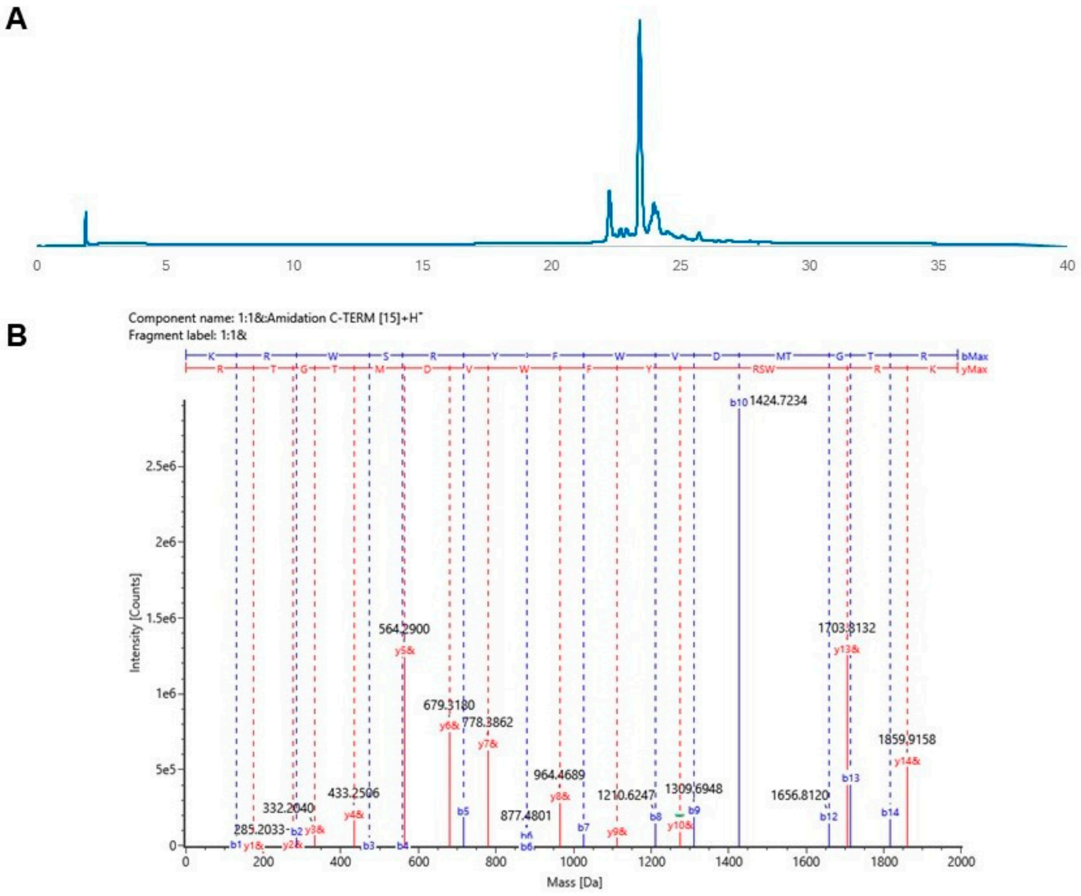

Figure S10. (**A**) HPLC chromatogram and (**B**) MS/MS spectrum of OB8 peptide.

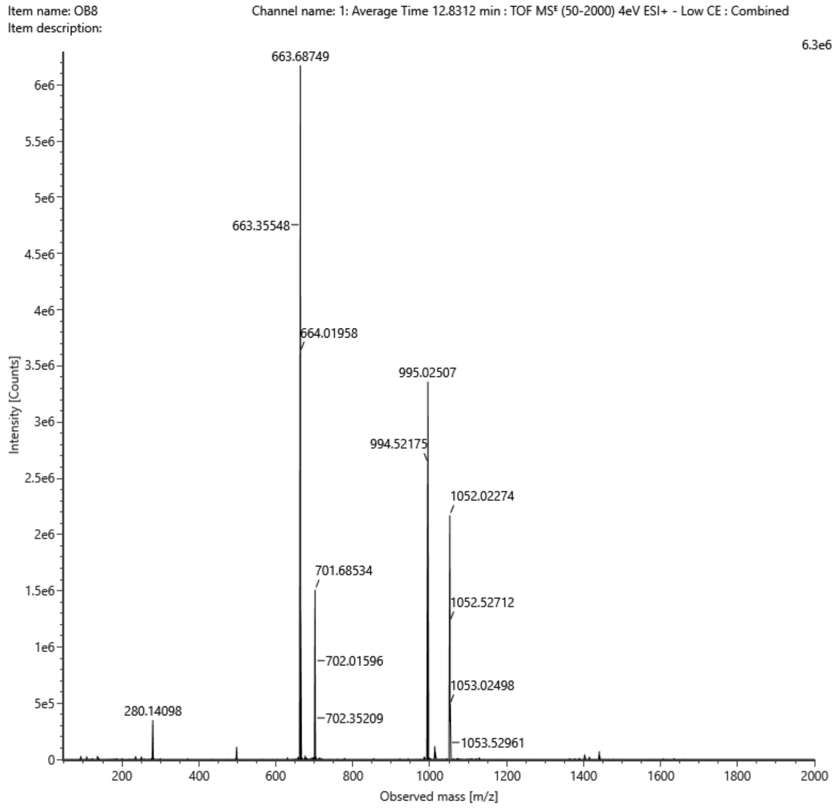

Figure S11. MS Spectra of OB8 peptide.
